# Evaluation of Dispersion Behavior and Practicality of PGPR@ZnO Nano-Hyperdispersant in DEHC

**DOI:** 10.3390/nano16080455

**Published:** 2026-04-12

**Authors:** Rui Zhang, Patiman Abudu, Xiaoqing Li, Wumanjiang Eli

**Affiliations:** 1 College of Chemistry and Chemical Engineering, Xinjiang Normal University, Urumqi 830054, China; 15276681225@163.com; 2 College of Chemical Engineering, Xinjiang Vocational University, Urumqi 830013, China; 3 Key Laboratory of Chemistry and Chemical Engineering on Heavy-Carbon Resources, School of Chemistry and Chemical Engineering, Yili Normal University, Yining 835000, China; patiman211@163.com; 4 Xinjiang Jinxuechi Technology Co., Ltd., Urumqi 830026, China; jinxuechi2023@163.com

**Keywords:** nanofluid, dispersion stability, nano-hyperdispersant, thermal conductivity, di(2-ethylhexyl) carbonate, zinc oxide, polyglycerol polyricinoleate, immersion coolant

## Abstract

To achieve stable dispersion of ZnO nanoparticles in the base fluid and enhance thermal conductivity (λ), a PGPR@ZnO nano-hyperdispersant was synthesized using polyglycerol polyricinoleate (PGPR) and ZnO. FT-IR and DSC confirmed the bonding interaction between PGPR and ZnO, and zeta potential analysis verified the steric hindrance effect that effectively inhibits particle agglomeration. The PGPR@ZnO was dispersed into di(2-ethylhexyl) carbonate (DEHC) by ultrasonication and stirring, yielding a stable DEHC-PGPR@ZnO nanofluid. This nanofluid achieved a 16.2% increase in λ while retaining the low viscosity and low pour point of the base fluid. Stability assessments showed consistent particle size main peaks before and after static and dynamic tests, with no obvious agglomeration peaks, average particle size variation below 6%, PDI below 0.3, and negligible zeta potential fluctuation. Following static and dynamic stability tests, the thermal conductivity decreased by 0.85% and 7.98%, respectively. These results indicate excellent dispersion stability and provide a valuable reference for evaluating the operational adaptability of the coolant. The nanofluid meets the basic standards for immersion coolants and exhibits a figure of merit (FOM) superior to most oil-based coolants. Compared with PAO2, it offers advantages in raw material availability and resistance to hydrolysis and acidification, providing research and practical foundation for the development of high-performance immersion coolants.

## 1. Introduction

With the accelerated pace of digital transformation and upgrading across all industries, the application demand for data centers has been greatly driven, leading to a rise in energy consumption and a growth in thermal management requirements [1,2]. Reports from the International Energy Agency (IEA) indicate that global electricity consumption by data centers grew from approximately 205 terawatt-hours (TWh) in 2018 to 415 TWh in 2024, and is projected to reach 945 TWh by 2030, accounting for 3–5% of global total electricity consumption [3,4]. Therefore, the development of stable, high-efficiency heat transfer media is of crucial importance.

In the base fluid, dispersed nanoparticles can enhance the microscale convection and the interactions between particles can boost the thermal conductivity (λ) of the base fluid, thereby achieving higher thermal conversion efficiency [5,6,7]. Eastman et al. [8] dispersed copper nanoparticles in ethylene glycol base fluid to fabricate nanofluids; when approximately 0.3 *v*/*v*% of copper nanoparticles were added, the effective λ of the mixture rose by 40%. Chon et al. [9] prepared alumina nanofluids with different concentrations and particle sizes, and determined λ of the fluids in the temperature range of 21 °C to 71 °C. The experimental results showed that the nanoparticles in the fluid can effectively enhance λ of nanofluids [10].

The stability of nanoparticles in base fluids is the key factor determining heat transfer performance. Due to their small particle size and large specific surface area, nanoparticles in base fluids exhibit increased interactions with one another, and tend to agglomerate under the attraction of van der Waals forces, resulting in reduced stability in the fluids [11,12]. Yang et al. [13] investigated the thermal stability and thermal performance of oil-based CuO nanofluids for solar thermal applications, and verified that stability was positively correlated with λ. Lv et al. [14] demonstrated that oleic acid-functionalized TiO_2_ exhibited the optimal dispersion stability when dispersed in transformer oil via a combined method of stirring and ultrasonic bath. Moreover, excessive agglomeration of nanoparticles leads to the deterioration of the overall rheological properties and inhibits efficient heat transfer under long-term stability conditions. Therefore, the high λ efficiency of nanofluids is highly dependent on the stability of the system, and how to achieve the long-term stability of nanofluids has become a top priority in both basic research and practical applications [15].

Di(2-ethylhexyl) carbonate (DEHC) contains both polar ester functional groups (-O-C(=O)-O-) and nonpolar long-chain alkyl structures in its molecular structure, which endows it with excellent compatibility with materials such as metals, plastics, and rubbers [16,17,18]. PAO2 is a commonly used material in the field of oil-based immersion cooling. However, during long-term service under conditions such as high temperature and coexistent water vapor, it undergoes synergistic degradation via hydrolysis and thermal oxidation. The tertiary carbon structures in its molecular chains are prone to cleavage and subsequent reaction with oxygen, generating small-molecular organic acids, which in turn leads to an elevated acid value and increased maintenance costs [19]. Compared with PAO2, DEHC has low viscosity and low pour point comparable to those of PAO2, while avoiding the drawbacks of PAO2 such as hydrolysis-induced formation of organic acids, scarce production, and limited accessibility. These characteristics make DEHC hold potential application prospects in the field of immersion coolants.

Zinc oxide (ZnO) can enhance λ of base oils; however, it is one of the nanoparticles with the highest density, making it a challenge to ensure the stable dispersibility of ZnO in base oils [10]. As a highly efficient hydrophobic emulsifier, polyglycerol polyricinoleate (PGPR) possesses long hydrophobic tails (ricinoleoyl groups) and hydrophilic heads (polyglycerol groups) on its molecular chains. These structural features enable it to spontaneously form micelle or vesicle structures under specific conditions, rapidly reduce the oil-water interfacial tension, and prevent nanoparticles from aggregating or settling during storage [20,21,22].

Based on this, the PGPR@ZnO nano-hyperdispersant material was prepared and further dispersed in the di(2-ethylhexyl) carbonate (DEHC) base fluid via a combined ultrasonication and stirring method in this study, affording a stable DEHC-PGPR@ZnO nanofluid. Ultraviolet-visible spectrophotometry (UV–Vis) and differential scanning calorimetry (DSC) were employed to evaluate the regulatory effect of PGPR on the dispersion performance of ZnO. Combined with particle size and zeta potential analyses, as well as static and dynamic stability tests, the excellent dispersion stability of the DEHC-PGPR@ZnO nanofluid was verified. Additionally, the performance of the as-prepared nanofluid was tested, and the results confirmed that it meets all the basic performance requirements for oil-based immersion coolants. To objectively and quantitatively assess its application potential, the Figure of Merit (FOM) was introduced for a comparative analysis with the oil-based immersion coolants reported in the existing literature, which demonstrated that the prepared nanofluid exhibits distinct performance advantages. This study not only develops a highly stable nanofluid but also provides a theoretical and practical foundation for the design and development of high-performance oil-based immersion coolants in the field of thermal management.

## 2. Materials and Methods

### 2.1. Materials

All reagents were purchased from commercial suppliers and were ready for use without further purification. Zinc oxide nanoparticles (average particle size: ca. 50 ± 10 nm, purity ≥ 99.9%) were acquired from Macklin. Polyglycerol polyricinoleate (PGPR, diglycerol, triglycerol and tetraglycerol ≥ 75 wt%, polyglycerol consisting of ≥7 glycerol units ≤ 10 wt%) was obtained from Adamas (Tansoole Platform, Shanghai, China). Di(2-ethylhexyl) carbonate (DEHC) was supplied by Lulian Jining Chemical Technology Co., Ltd (Jining, China). Hydrochloric acid (HCl, AR) was procured from Sinopharm Chemical Reagent Co., Ltd (Shanghai, China). Sodium hydroxide (NaOH, AR) was provided by Tianjin Beilian Fine Chemicals Development Co., Ltd (Tianjin, China). Borax (Na_2_B_4_O_7_·10H_2_O, AR) was purchased from General-Reagent(Tansoole Platform, Shanghai, China). Boric acid (H_3_BO_3_, AR) was supplied by Tianjin Zhiyuan Chemical Reagent Co., Ltd (Tianjin, China). Zinc monosodium salt (99%) was obtained from Damas-beta (Tansoole Platform, Shanghai, China). Zn standard solution (certified reference material, 1 mg/mL) was purchased from the National Institute of Metrology, China (NIM, Beijing, China).

### 2.2. Preparation of PGPR@ZnO Nano-Hyperdispersant

ZnO nanoparticles were added to PGPR in batches and ground to a paste-like state, yielding the PGPR@ZnO nano-hyperdispersant with a ZnO mass fraction of 80%. The structure of the product was analyzed utilizing a TENSOR 27 Fourier-transform infrared spectrometer (FT-IR, Bruker Corporation, Berlin, Germany). The interaction between PGPR and ZnO was investigated by differential scanning calorimeter (DSC, DSC3, METTLER-TOLEDO, greifensee, Switzerland) under a nitrogen atmosphere from 50 °C to 500 °C at a heating rate of 10 °C·min^−1^.

### 2.3. Preparation of DEHC-PGPR@ZnO Nanofluid

A predetermined amount of PGPR@ZnO was weighed and added into DEHC, separately, yielding final ZnO concentrations of 0.5, 1.0, 2.5, 5.0, 10.0, and 15.0 mg/mL, respectively. The mixture was first ultrasonicated for 1 h (ultrasonic power: 480 W, Shanghai Shangpu Instrument & Equipment Co., Ltd., Shanghai, China) followed by magnetic stirring for 2 h at a rotation speed of 520 rpm to obtain DEHC-PGPR@ZnO nanofluids. Meanwhile, a DEHC-ZnO system without PGPR was prepared under the same conditions as a control sample.

### 2.4. Evaluation of Dispersion Effect

The nanofluids with different ZnO concentrations were prepared by static (24 h) and centrifugal (10,000 rpm, 10 min) methods. First, 1 mL of the supernatant of nanofluid was taken and placed in a crucible, which was then calcined in a muffle furnace until ashed. Subsequently, 5 drops of 6 mol/L dilute HCl were added to dissolve the ash, followed by the addition of 10 mL boric acid–borax buffer solution (pH = 9) and 3 mL zinc reagent. The resulting solution was transferred into a 50 mL volumetric flask, and its pH was adjusted to 9 with sodium hydroxide solution before being diluted to the mark with distilled water. DEHC treated with the same method served as a blank control. The absorbance was measured at 620 nm using a UV–visible spectrophotometer (UV–Vis, TU-1901, Beijing Purkinje General Instrument, Beijing, China) [23], with all measurements conducted in triplicate. If the absorbance of the sample exceeded the linear range of the calibration curve, the sample was diluted before measurement. Based on the average absorbance value and Zn standard curve (Figure S1), the residual ZnO content and utilization rate in the nanofluids were calculated. The dispersion stability of the nanofluids was then evaluated by analyzing the changes in ZnO residual amount and utilization efficiency before and after centrifugation, and the optimal ZnO concentration with favorable dispersibility and anti-sedimentation performance in the DEHC system was screened out.

### 2.5. Evaluation of Dispersion Stability

After one centrifugation cycle, the DEHC-PGPR@ZnO nanofluid prepared at the optimal ZnO concentration was subjected to both static and dynamic stability tests synchronously. Both tests were conducted via UV–Vis spectral scanning within the wavelength range of 200–450 nm. Samples were taken and tested every 5 days, and the dispersion state of ZnO nanoparticles was determined according to the shift or intensity change in the spectral curves. The static stability test was conducted under the following conditions: 300 mL of the nanofluid was left to stand for 30 days at room temperature (20–25 °C). For the dynamic stability test, a peristaltic pump fitted with silicone tubing was adopted to simulate practical working conditions via circulation; the test was performed with 300 mL of the nanofluid at a controlled temperature of 40 °C and a circulation flow rate of 10 mL/min, with continuous operation for 30 days.

The zeta potential of nanofluids and the particle size of nanoparticles were determined using a nanoparticle size and zeta potential analyzer (ZS90, Malvern Instruments, Worcestershire, UK), with all measurements conducted in triplicate.

### 2.6. Evaluation of Nanofluids Performance and Applications

#### 2.6.1. Basic Performance

The acid value (AV) and boiling point (b.p.) of nanofluids were ascertained in accordance with the GB/T 264-1983 petroleum-products determination of acid number [24] and SH/T 0089-1991 engine-coolant boiling-point determination method [25], respectively. The density (ρ) was measured by electronic densitometer (LC-LMH 3002, Shanghai Lichen Bangxi Instrument Technology, Shanghai, China). The flash point (FP) was measured by an open-cup flash point tester (AKD-K106, Yangzhou Ackeruid Instrument Co., Ltd., Yangzhou, China). The pour point (PP) was tested with a petroleum product pour point tester (SYD-510D, Shanghai Changji Geological Instrument Co., Ltd., Shanghai, China). The dielectric constant (ε) was characterized via an insulating oil dielectric loss and resistivity tester (HGJD203, HUIGONG ELECTRIC, Zibo, China).

The kinetic viscosity (ν) of the nanofluids at 40 °C was determined using an automatic viscosity tester (ELB-KVD10, INLAB Technology (Shanghai) Co., Ltd., Shanghai, China). Dynamic viscosity (μ) is calculated using the following formula:

(1) μt = ν_t_ρ_t_

where μ_t_ denotes the dynamic viscosity (mPa·s), ν_t_ represents the kinematic viscosity (mm^2^·s^−1^), and ρ_t_ indicates the density (g·m^3^), all measured at temperature t °C.

#### 2.6.2. Thermodynamic Property

The thermal conductivity (λ) of the nanofluid was measured at 25 °C using a LAMBDA thermal conductivity analyzer (Flucon Fluid Control GmbH, Bad Lauterberg, Germany), and the specific heat capacity (Cp) was determined by a differential scanning calorimeter (HNB-DSC 300C, Xiamen Yutisi Instrument Co., Ltd., Xiamen, China).

All measurements on the basic and thermodynamic properties of the nanofluids were performed in triplicate.

#### 2.6.3. Evaluation of Applications

To evaluate the potential application of DEHC-PGPR@ZnO nanofluids in the field of immersion cooling fluids, the concept of Figure of Merit (FOM) was introduced to assess the nanofluid. This experiment simulated the operation of a pump-driven immersion liquid-cooled cabinet, while the flow state inside the cabinet is usually turbulent under actual working conditions. Thus, the FOM can be expressed as follows [26,27]:

(2) FOM = λ^0.6^Cp^0.4^ρ^0.8^μ^−0.4^

where λ (W/(m·°C)) denotes the thermal conductivity of the fluid, Cp (J/(kg·°C)) represents the specific heat capacity, ρ (kg/m^3^) indicates the density, and μ (mPa·s) denotes the dynamic viscosity. It can be seen from the above equation that increases in λ, Cp and ρ contribute to the enhancement of the heat capacity of immersion cooling fluids, while the opposite is true for the increase in μ.

## 3. Results

### 3.1. Evaluation of Dispersion Stability

**Figure 1:**
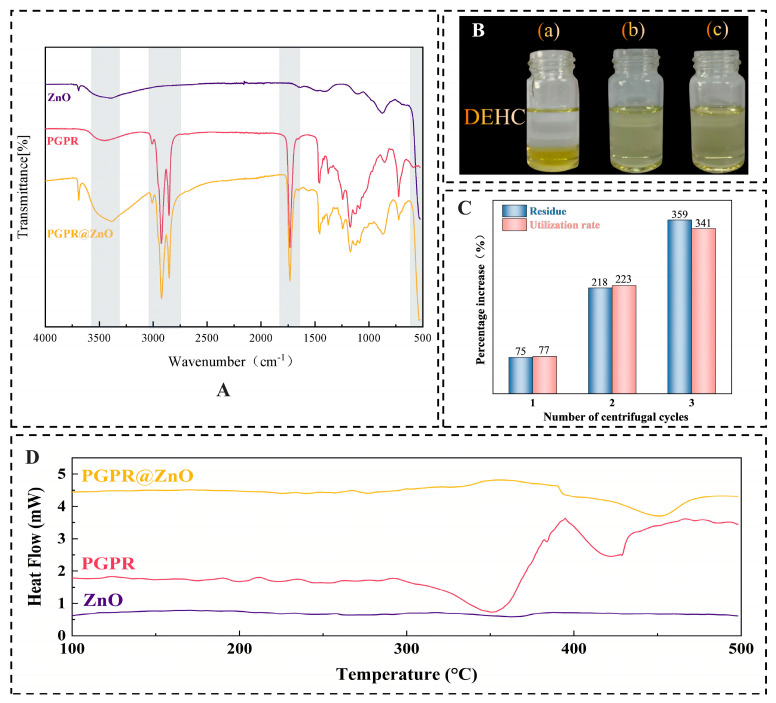
(**A**) FT-IR of ZnO, PGPR, and PGPR@ZnO. (**B**) Compatibility testing of PGPR with DEHC: (**a**) initial mixture; (**b**) ultrasonic stirring for 1 h followed by 2 h; (**c**) mixture left to stand for 24 h. (**C**) Percentage increase in residual content and utilization rate at the optimal addition rate of ZnO. (**D**) The DSC graph of ZnO, PGPR, and PGPR@ZnO.

To improve the dispersion effect of ZnO nanoparticles in DEHC and enhance the utilization rate of ZnO, this study prepared the nano-hyperdispersant material PGPR@ZnO and dispersed it into DEHC. To verify the successful preparation of PGPR@ZnO, FT-IR was employed to characterize the compositions of ZnO, PGPR, and PGPR@ZnO, as shown in Figure 1A. The peak at 524 cm^−1^ corresponds to the Zn-O stretching vibration peak of ZnO nanoparticles. The peaks at 1740 cm^−1^, 2850 cm^−1^, and 3400 cm^−1^ are assigned to the C=O stretching vibration peak of the carbonyl group in ester bonds, the C-H stretching vibration peak of alkyl chains, and the O-H stretching vibration peak of hydroxyl groups in polyglycerol of PGPR, respectively. All these characteristic peaks appeared in the PGPR@ZnO composite, confirming the successful preparation of the nano-hyperdispersant material PGPR@ZnO.

PGPR is a non-ionic surfactant with a structure combining long-chain alkyl groups and polyhydroxy polyethers; the long-chain alkyl groups are lipophilic, while the polyether terminals are hydrophilic [28]. As shown in Figure 1B, the amphiphilic property of PGPR enables its stable compatibility with DEHC.

To further verify the interfacial interaction, DSC analysis was performed under a nitrogen atmosphere from 100 °C to 500 °C at a heating rate of 10 °C/min (Figure 1D). ZnO maintains a flat baseline throughout the test range, confirming its excellent thermal stability. In contrast, pure PGPR exhibits a sharp exothermic peak at approximately 380 °C, corresponding to its oxidative decomposition process. Notably, the decomposition process of the PGPR@ZnO composite is significantly delayed, the sharp exothermic peak disappears, and its thermal behavior shifts to higher temperatures with a broadened peak profile. This result indicates that the interaction between PGPR and ZnO is not merely physical, and the chemical bonding between them restricts the molecular motion of PGPR chains, thereby improving its thermal stability.

As depicted in Figure S2, the DEHC-PGPR@ZnO nanofluid obtained by dispersing the PGPR@ZnO nano-superdispersed material in DEHC still exhibits a distinct color after three cycles of high-speed centrifugation, indicating that the incorporation of PGPR@ZnO can significantly improve the dispersion effect of ZnO in DEHC. As presented in Figure S3, taking the residual amount and utilization rate of ZnO into comprehensive consideration, the optimal ZnO loading concentration is determined to be 1 mg/mL, and all subsequent experiments are conducted on the system with this optimal loading concentration.

As illustrated in Figure 1C, compared with the system without PGPR, the ZnO utilization efficiency in the three centrifugation cycles of DEHC-PGPR@ZnO nanofluid is increased by 77%, 223%, and 341% respectively. This indicates that pristine ZnO exhibits poor dispersion stability in DEHC, and its utilization efficiency decreases drastically with an increase in the number of centrifugation cycles. The introduction of PGPR enhances the stability of ZnO in the base fluid, thereby leading to a substantial improvement in its utilization efficiency.

**Figure 2:**
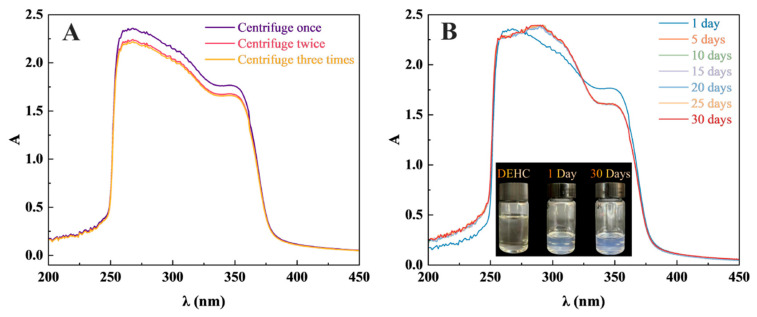
(**A**) UV–Vis absorption spectra of DEHC-PGPR@ZnO (1.0 mg/mL) after one, two, and three centrifugations; (**B**) UV–Vis absorption spectra of DEHC-PGPR@ZnO (1.0 mg/mL) after standing for different numbers of days. The photographs show comparisons of DEHC, DEHC-PGPR@ZnO (1.0 mg/mL) after standing for 1 day, and DEHC-PGPR@ZnO (1.0 mg/mL) after standing for 30 days.

To further validate the dispersion stability of DEHC-PGPR@ZnO nanofluids, the samples were subjected to high-speed centrifugation for three consecutive centrifugations. After each cycle, the nanofluids were characterized by UV–Vis absorption spectroscopy. As shown in Figure 2A, the characteristic intrinsic band gap absorption peak of ZnO appears at 370 nm [29]. The peak shapes remain consistent after three centrifugation cycles, indicating that the nanoparticles in the system are uniform in size and no severe agglomeration occurs. Compared with the state after one centrifugation cycle, the absorbance change after the second centrifugation cycle is less than 6%, and after the third centrifugation cycle it is less than 7.5%, demonstrating that DEHC-PGPR@ZnO possesses favorable stability and anti-sedimentation capability. This confirms that the effective coating of the PGPR modification layer on the ZnO surface enhances the steric hindrance effect between particles, improves their compatibility with the medium, and ensures that the composite material can maintain a uniform dispersion even after multiple centrifugation treatments.

Subsequently, the DEHC-PGPR@ZnO nanofluids were statically placed at room temperature (20–25 °C) for 30 days (static stability test). As shown in Figure 2B, the color of the long-term static DEHC-PGPR@ZnO nanofluid showed no obvious change. Samples were taken every 5 days and characterized by UV–Vis absorption spectroscopy. The results demonstrated that the position and intensity of the absorption peak of the DEHC-PGPR@ZnO nanofluids exhibited no noticeable changes within 30 days. This further indicates that the coating formed by PGPR on the surface of ZnO effectively prevents particle agglomeration and maintains long-term dispersion, which basically meets the requirements for long-term application as immersion coolants.

**Figure 3:**
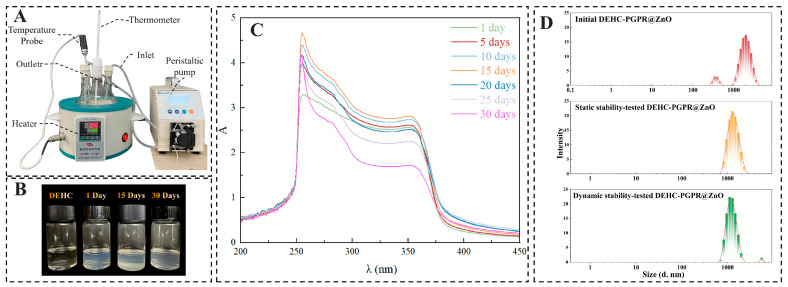
DEHC-PGPR@ZnO (1.0 mg/mL): (**A**) Schematic of the dynamic stability testing apparatus; (**B**) comparative status after 1 day, 15 days, and 30 days of cycling; (**C**) UV–Vis absorption spectra after different cycling durations; (**D**) particle size distribution diagrams of DEHC-PGPR@ZnO (1.0 mg/mL) before and after static and dynamic tests.

The results of the concurrent dynamic stability tests (Figure 3A–C) indicate that the absorbance at 370 nm of the DEHC-PGPR@ZnO nanofluid increased by 8.2% after continuous operation for 15 days under simulated working conditions at 40 °C. This suggests that the circulation of the peristaltic pump enhanced the encapsulation of ZnO nanoparticles by PGPR at the initial stage of operation, leading to a more uniform dispersion of the PGPR@ZnO nano-hyperdispersant in the base fluid. After 30 days of continuous operation, the absorbance dropped by 33%, accompanied by a small amount of precipitation at the bottom of the bottle. This demonstrates that the shear force generated by the peristaltic pump circulation damaged the structure of the PGPR@ZnO nano-hyperdispersant or induced its rearrangement, thus resulting in a decline in stability.

As shown in Figure 3D, the main peak positions of the particle size distributions of the DEHC-PGPR@ZnO nanofluid are consistent before the test, after the static test and after the dynamic test, and no obvious large-size particle aggregation peak appears, indicating that the nanofluid maintains good dispersion stability without significant aggregation under static placement and dynamic flow [30].

As listed in Table 1, the initial DEHC-PGPR@ZnO nanofluid has an average particle size of 1497 nm, a polydispersity index (PDI) of 0.243, and a zeta potential of −0.0363 mV. After the static stability test, the average particle size slightly increases to 1566 nm (4.6% increment), the PDI rises slightly to 0.264, and the zeta potential changes to −0.0434 mV. After the dynamic stability test, the average particle size slightly decreases to 1417 nm (5.3% decrement), the PDI reduces to 0.197, and the zeta potential changes to −0.0221 mV. These results demonstrate that the interaction between nanoparticles in the nanofluid is not simple electrostatic repulsion. The steric hindrance effect formed by PGPR molecules on the ZnO surface effectively prevents particle aggregation, and the introduction of PGPR can significantly improve the dispersion stability of ZnO in DEHC.

### 3.2. Evaluation of Nanofluids Performance and Applications

#### 3.2.1. Basic Performance

As an ester-based base oil, DEHC exhibits excellent chemical stability. It does not produce corrosive acid upon hydrolysis and is less prone to saponification compared with other esters, thus featuring a favorable environmental friendliness [31,32]. Table 2 compares the basic properties and thermodynamic properties of DEHC base fluid and DEHC-PGPR@ZnO nanofluids. The results show that at 40 °C, the low dynamic viscosity decreased by 0.8%, FP increased by 10%, b.p. and PP exhibited no obvious change, and AV slightly increased, indicating that the addition of the PGPR@ZnO nano-hyperdispersant does not change the basic properties of the base fluid. The data demonstrate that the ultra-low viscosity of the nanofluid prepared in this study renders it unrestricted by operating modes. Its high FP displays relatively stable chemical properties, posing low risks under conventional industrial heating or storage conditions. Additionally, the ultra-low pour point enables it to maintain excellent fluidity in low-temperature environments, while the low AV makes it resistant to hydrolysis or oxidation reactions, and thus less prone to deterioration during storage and application [33].

#### 3.2.2. Thermodynamic Property

**Figure 4:**
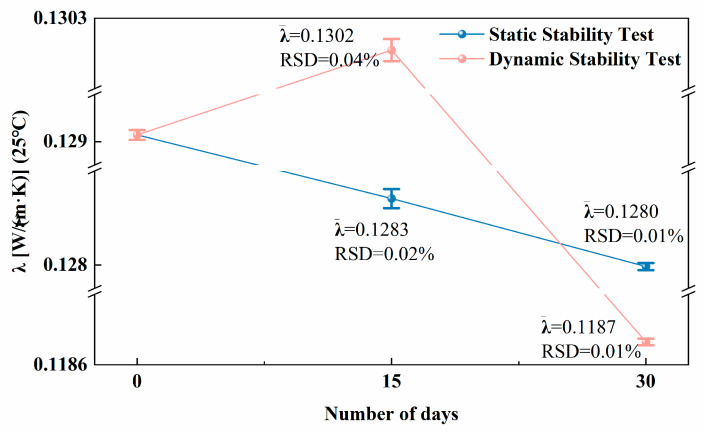
Thermal conductivity of nanofluids during static and dynamic stability testing procedures over different days (to clearly visualize errors, set three breakpoints when plotting the vertical axis).

Thermodynamic data demonstrate that the average λ of DEHC is 0.111 W/(m·K), and that of DEHC-PGPR (1.0 mg/mL) is 0.112 W/(m·K), which indicates that the incorporation of PGPR has no significant effect on the thermal conductivity of DEHC. Compare with DEHC, λ of the DEHC-PGPR@ZnO nanofluid is augmented by 16.2% and its specific heat capacity by 2.5%, implying that the introduction of the PGPR@ZnO nano-hyperdispersant material optimizes the base fluid in terms of heat transfer, heat storage, and temperature uniformity. To further verify the thermal conductivity performance and long-term stability of the nanofluids prepared in this study, the nanofluids used in static and dynamic stability tests were sampled every 15 days for λ measurement. As shown in Figure 4, compared with the initial DEHC-PGPR@ZnO nanofluid, its λ decreased by 0.54% after 15 days of static storage and by 0.85% after 30 days. This indicates that ZnO nanoparticles, wrapped by PGPR, remain stably dispersed during the static storage process without obvious agglomeration, thereby ensuring the relative stability of λ. In dynamic conditions, λ of the nanofluid increased by 0.93% after 15 days of continuous operation and decreased by 7.98% after 30 days, which is consistent with the trend observed in UV–Vis measurements. This further confirms that the circulation of the peristaltic pump exerts a positive effect on the PGPR@ZnO nano-hyperdispersant at the initial stage of the dynamic stability test, whereas the shear force disrupts the PGPR@ZnO coating layer to a certain extent, causing sedimentation and a subsequent reduction in thermal conductivity.

#### 3.2.3. Evaluation of Applications

The as-prepared DEHC-PGPR@ZnO nanofluid, while retaining the excellent properties of the DEHC base fluid, demonstrates significantly enhanced λ through the incorporation of PGPR@ZnO nano-hyperdispersant. Moreover, its outstanding stability makes it a promising candidate for applications in immersion coolants. Specifically, immersion coolants are required to exhibit outstanding thermophysical properties, material compatibility, chemical and thermal stability, non-corrosiveness, and environmental friendliness [34]. Meanwhile, they should comply with the following standards: Cp > 960 J/(kg·K), λ > 0.06 W/(m·K), ε < 2.3, b.p. > 100 °C, and FP > 150 °C for oil-based coolants [26]. Based on the comprehensive performance parameters, the Cp of DEHC-PGPR@ZnO nanofluids is 1851 J/(kg K), λ is 0.129 W/(m K), ε is 2.15, b.p. is 298 °C and FP is 152 °C, which completely satisfy the fundamental requirements for immersion coolants.

To further evaluate the potential application of the DEHC-PGPR@ZnO nanofluid in immersion cooling systems, the FOM value was introduced for assessment. Saylor et al. [35] first proposed a figure of merit (FOM) for assessing and comparing the performance of some candidate immersion coolants and qualitatively analyzed the relationship between the performance of pump-driven immersion coolants and their thermophysical properties such as density, specific heat capacity, and the viscosity of coolants. Chen et al. [27] established a comprehensive measurement platform for the thermophysical properties of electronic fluorinated liquids, derived the FOM suitable for single-phrase immersion coolants in data centers, and quantitatively determined the influence weight of thermal properties on performance, with μ > λ > ρ > Cp. Moreover, numerical verification confirmed that electronic fluorinated liquids with low dynamic viscosity can significantly enhance heat transfer performance and reduce flow pressure drop. Compared with fluorinated fluids reported in the literature [36,37,38,39], DEHC-PGPR@ZnO nanofluids exhibit not only low viscosity but also high thermal conductivity, giving them a distinct advantage over most fluorinated fluids.

Based on the above, the FOM value of DEHC-PGPR@ZnO nanofluids was calculated and compared with that of oil-based coolants reported in the literature, where a higher FOM value indicates better thermal performance of the coolant. As shown in Table 3 [26], the FOM value of DEHC-PGPR@ZnO nanofluids is significantly superior to that of silicone oil, mineral oil, PAO4, and PAO6. Although the FOM value of PAO2 is slightly higher than that of the DEHC-PGPR@ZnO nanofluids, low-viscosity PAO2 is predominantly imported, resulting in limited availability. Additionally, prolonged use of PAO2 leads to hydrolysis, generating small-molecule organic acids that increase maintenance costs. In contrast, DEHC-PGPR@ZnO nanofluids demonstrate significant advantages, with its base fluid derived from localized raw materials and processes, thus possessing significant cost advantages. Moreover, as a carbonate compound, DEHC hydrolyzes without generating organic acids, demonstrating strong compatibility and environmental friendliness. These characteristics highlight its competitiveness as an immersion coolant, which is therefore expected to be widely applied in fields such as data centers and high-performance computing.

## 4. Conclusions

In summary, the PGPR@ZnO nano-hyperdispersant material was prepared and further dispersed in DEHC base fluid via the ultrasound and stirring method, yielding a stable DEHC-PGPR@ZnO nanofluid. FT-IR analysis results confirmed the formation of effective bonding interactions between PGPR and ZnO nanoparticles, which laid a structural foundation for enhancing dispersion stability. The dispersion effect of different ZnO addition concentrations in the base fluid was investigated to select the optimal concentration, at which DEHC-PGPR@ZnO nanofluids were prepared. Compared with the DEHC-ZnO system, after three centrifugation cycles, the utilization rates of ZnO in DEHC-PGPR@ZnO nanofluids increased by 77%, 223%, and 341% respectively, demonstrating that PGPR modification significantly improves the dispersion efficiency of ZnO nanoparticles in DEHC.

The results of stability test showed that the UV–Vis peak profile of the DEHC-PGPR@ZnO nanofluid remained consistent after three cycles of high-speed centrifugation. During the static stability period, the position and intensity of the UV–Vis absorption peak of the nanofluid showed no significant changes, and the particle size increased by 4.6%. During the dynamic stability process, the UV–Vis peak intensity exhibited a trend of first increasing and then decreasing, reaching the maximum on the 15th day. A small amount of precipitation was observed at the end of operation, and the average particle size decreased by 5.3%. This indicated that the shear force of the peristaltic pump could improve the mixing of the nano-hyperdispersant with the base fluid at the initial stage of operation, while prolonged operation would damage the PGPR@ZnO structure and induce sedimentation. The variation range of the average particle size was <6%, PDI < 0.3, and the zeta potential fluctuated slightly in both processes. This demonstrates that the interaction between nanoparticles in the nanofluid is not simple electrostatic repulsion. PGPR molecules form a steric hindrance effect on the ZnO surface, which effectively inhibits particle agglomeration. The introduction of PGPR can significantly improve the dispersion stability of ZnO in DEHC.

The basic performance test shows that the DEHC-PGPR@ZnO nanofluid retained the low viscosity and low pour point characteristics of the original DEHC base fluid. In addition, thermodynamic tests revealed that the base fluid incorporated with the PGPR@ZnO nano-hyperdispersant achieved a 16.2% increase in **λ**. Following static and dynamic stability tests, the thermal conductivity decreased by 0.85% and 7.98%, respectively. These results provide a valuable reference for evaluating the operational adaptability of the coolant.

Furthermore, the performance data demonstrate that the DEHC-PGPR@ZnO nanofluid meets the basic requirements for immersion coolants. Meanwhile, the FOM value indicates that the nanofluid is superior to silicone oil, mineral oil, PAO4 and PAO6, and had the advantages of easy raw material acquisition and less hydrolysis acid production compared with PAO2.

In summary, the DEHC-PGPR@ZnO nanofluid prepared in this study not only retains the low viscosity and low pour point of the DEHC base fluid but also exhibits outstanding dispersion stability and excellent thermodynamic properties. This work provides valuable research references and practical approaches for the innovative development of high-performance immersion coolants.

## Figures and Tables

**Table 1:** Average particle size, PDI and zeta potential of DEHC-PGPR@ZnO before and after static and dynamic stability tests.

Sample	Average Particle Diameter (nm)	PDI	Zeta Potential (mV)	RSD (%)
Initial	1497	0.243	−0.0363	8.82
After static stability tests	1566	0.264	−0.0434	9.77
After dynamic stability Tests	1417	0.197	−0.0221	13.6

**Table 2:** Performance parameters of DEHC and DEHC-PGPR@ZnO.

Performance Parameters	DEHC	DEHC-PGPR@ZnO
AV mg/KOH	0.06	0.10
ρ kg/m^3^	867	854
b.p. °C	302	298
μ (40 °C) mPa·s	3.81	3.78
FP °C	138	152
PP °C	<−70	<−70
ε	2.03	2.15
λ W/(m·K)	0.111	0.129
C_p_ J/(kg·K)	1805	1851

**Table 3:** Comparison of immersion coolant performance parameters between this work and the literature.

Name	ρ (25 °C)	μ (35 °C)	Cp	λ	FOM	References
	kg/m^3^	mPa·s	J/(kg·K)	W/(m·K)		
Silicone oil	957	42	1491	0.143	315	[27,40]
Mineral oil	849	11	2198	0.130	539	[38,41]
PAO2	796	4.1 (40 °C)	2300	0.150	843	[42]
PAO4	798	17	2143	0.150	465	[39]
PAO6	817	29	2377	0.145	391	[43]
This work	854	3.78 (40 °C)	1851	0.129	768	

## Data Availability

The original contributions presented in this study are included in the article. Further inquiries can be directed to the corresponding authors.

## Supplementary Materials

The following supporting information can be downloaded at: https://www.mdpi.com/article/10.3390/nano16080455/s1. Figure S1: Zn Standard Curve. Figure S2: Photographs of DEHC-ZnO (A) and DEHC-PGPR@ZnO (B) after treatment with 24 h static incubation, one centrifugation, two centrifugations, and three centrifugations, respectively. (a–g) Different ZnO addition concentrations: (a) 0 mg/mL; (b) 0.5 mg/mL; (c) 1.0 mg/mL; (d) 2.5 mg/mL; (e) 5 mg/mL; (f) 10 mg/mL; (g) 15 mg/mL. Figure S3: (a,b) Relationship between initial addition concentration and residual amount of ZnO nanoparticles after DEHC-ZnO and DEHC-PGPR@ZnO were left at rest for 24 h, centrifuged once, centrifuged twice, and centrifuged three times; (c,d) relationship between the initial addition concentration and utilization rate of ZnO nanoparticles in DEHC-ZnO and DEHC-PGPR@ZnO after 24 h of standing, one centrifugation, two centrifugations, and three centrifugations. Table S1: Spike recovery results. Table S2: ZnO residual amount and utilization rate at varying addition concentrations and centrifugation cycles. Reference [44] is cited in the supplementary materials.

## Abbreviations

The following abbreviations are used in this manuscript:

DEHC	Di(2-ethylhexyl) carbonate
ZnO	Zinc oxide
PGPR	Polyglycerol polyricinoleate
AV	Acid value
ρ	Density
b.p.	Boiling point
μ	Dynamic viscosity
FP	Flash point
PP	Pour point
ε	Dielectric constant
λ	Thermal conductivity
Cp	Specific heat capacity
FOM	Figure of Merit
FT-IR	Fourier-transform infrared spectrometer
UV–Vis	UV–visible spectrophotometer
DSC	Differential scanning calorimetry
