# Supplementary material for: Evaluation of Dispersion Behavior and Practicality of PGPR@ZnO Nano-Hyperdispersant in DEHC
Source: Nanomaterials (Basel). 2026 Apr 12;16(8):455. doi: 10.3390/nano16080455 (PMC13118329; doi:10.3390/nano16080455)
Supplement: Supplementary file 1 [file nanomaterials-16-00455-s001.zip › nanomaterials-4201302-supplementary.pdf]

Supporting information

Evaluation of Dispersion Behavior and Practicality of PGPR@ZnO Nano-Hyperdispersant in DEHC

Rui Zhang [1,2], Patiman Abudu [3], Xiaoqing Li [4] and Wumanjiang Eli [1,*]

[1] College of Chemistry and Chemical Engineering, Xinjiang Normal University, Urumqi 830054, China; 15276681225@163.com
[2] College of Chemical Engineering, Xinjiang Vocational University, Urumqi 830013, China
[3] Key Laboratory of Chemistry and Chemical Engineering on Heavy-Carbon Resources, School of Chemistry and Chemical Engineering, Yili Normal University, Yining 835000, China; patiman211@163.com
[4] Xinjiang Jinxuechi Technology Co., Ltd., Urumqi 830026, China; jinxuechi2023@163.com
* Correspondence: 13999116406@163.com

1. Solution Preparation

pH 9 buffer solution: Weigh 0.953 g of sodium tetraborate decahydrate, dissolve it in water, transfer the solution to a 100 mL volumetric flask, and add distilled water to the mark. Subsequently, weigh 0.309 g of boric acid, dissolve in water, transfer to a 100 mL volumetric flask, and add distilled water to the mark. After preparing both solutions, they were mixed thoroughly, and finely adjust the pH to 9 with 6 mol/L hydrochloric acid solution or 4% NaOH solution.

Zinc reagent: Weigh 0.13 g of zinc monosodium salt, dissolve in 2 mL of 4% NaOH solution, transfer to a 100 mL volumetric flask, and add distilled water to the mark.

Zn standard stock solution (0.1 mg/mL): Transfer 10 mL of 1 mg/mL Zn standard solution (GBW08620, National Institute of Metrology, China) into a 100 mL volumetric flask, dilute with water, and add distilled water to the mark.

Zn diluted standard solution (0.01 mg/mL): Transfer 10 mL of the Zn standard stock solution into a 100 mL volumetric flask, dilute with water, and add distilled water to the mark.

2. Preparation and Results of Zn Standard Curve

Accurately pipette 0, 0.10, 0.20, 0.30, 0.40 and 0.50 mL of the diluted zinc standard solution (0.01 mg/mL) into 50 mL volumetric flasks, respectively. Add 10 mL of boric

acid-borax buffer solution (pH = 9) and 3 mL of zincon reagent to each flask, then dilute to the mark with distilled water. The absorbance was measured at 620 nm by UV-Vis spectrophotometry with DEHC as the blank, and the calibration curve was plotted and fitted.

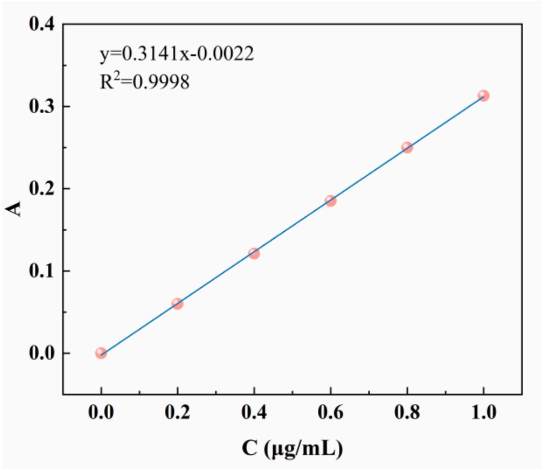

Figure S1 Zn Standard Curve

As shown in **Figure S1**, where y stands for absorbance and x stands for Zn concentration (μg/mL), the correlation coefficient R^2=0.9998, indicating a good linear relationship.

3. Experiment and Results Spiked recovery

The accuracy of the quantitative analytical method for ZnO in DEHC-PGPR@ZnO nanofluids was verified by a spike recovery experiment. Using DEHC-PGPR@ZnO (1.0 mg/mL) as the background sample, 0.1 mL and 0.3 mL of 1 mg/mL Zn standard solution were added separately. The samples were treated following Method 2.4, and the absorbance was measured at 620 nm with pure DEHC as the blank. All measurements were performed in triplicate.

The calculation equation for the spike recovery rate is as follows:

$$\text{Recovery (\%)}=(m_t-m_0)\cdot m_a^{-1}\cdot 100\%$$

Where, m_0 is the mass of ZnO in the background sample (mg), m_t is the total mass of ZnO in the spiked sample (mg), m_a is the mass of the spiked standard ZnO (mg).

According to the Zn standard curve, the average mass of the background sample was determined to be 0.678 mg, as shown in **Table S1**. The average recovery of the low-level spiked group was 97.6% (relative standard deviation, RSD=3.29%), and that of the high-level

spiked group was 98.1% (RSD=4.50%). The results indicating that the quantitative method exhibits good precision and accuracy, and can be applied for the reliable quantitative analysis of ZnO in nanofluids.

Table S1. Spike recovery results

Sample	Spiked mass (mg)	Average recovery (%)	SD	RSD (%)
**Low-level spiked sample**	0.1	97.6	0.0322	3.29
**High-level spiked sample**	0.3	98.1	0.0442	4.50

4. The calculation equations for the residual amount and utilization rate of ZnO

The calculation equation for the residual amount of ZnO is as follows:

$$m_{r\,(ZnO)}=aC_{Zn}\cdot V_l\cdot M_{Zn}^{-1}\cdot M_{ZnO}\cdot 10^{-3}$$

where $m_{r\,(ZnO)}$ is the residual amount of ZnO (mg); C_{Zn} is the Zn concentration derived from the zinc standard curve (μg/mL); V_l is the solution volume (mL); a is the dilution factor; and M_{Zn} and M_{ZnO} are the molar masses of Zn and ZnO, respectively (g/mol).

The calculation equation for the utilization rate of ZnO is as follows:

$$\omega_{ZnO}\ (\%)\ =m_{r\,(ZnO)}\cdot m_t^{-1}\cdot 100\%$$

where m_t is the total added amount of ZnO (mg).

5. Evaluation of dispersion effect

The dispersion behavior of ZnO nanoparticles in the base fluid is significantly affected by differences in polarity and intermolecular forces. As shown in **Figure S2A,** the color of the product gradually deepens with the increase in ZnO loading concentration; However, after standing for 24 h, the DEHC-ZnO suspension is relatively stable, the fluid becomes completely transparent following high-speed centrifugation.

As depicted in **Table S2, Figure S3(a) and (c)**, the residual amount of ZnO in DEHC-ZnO decreases with increasing centrifugation cycles, and the maximum utilization rate of ZnO in

DEHC does not exceed 10%, indicating that the dispersion stability of ZnO nanoparticles in DEHC is not ideal. Although the polar carbonate groups in DEHC molecules can form hydrogen bonds or coordination bonds with hydroxyl groups or oxygen ions on the ZnO surface, thereby reducing the interfacial energy, this interaction is insufficient to provide adequate steric hindrance for stabilizing the ZnO surface[1]. Consequently, ZnO particles attract each other via van der Waals forces, forming large aggregates that eventually sediment.

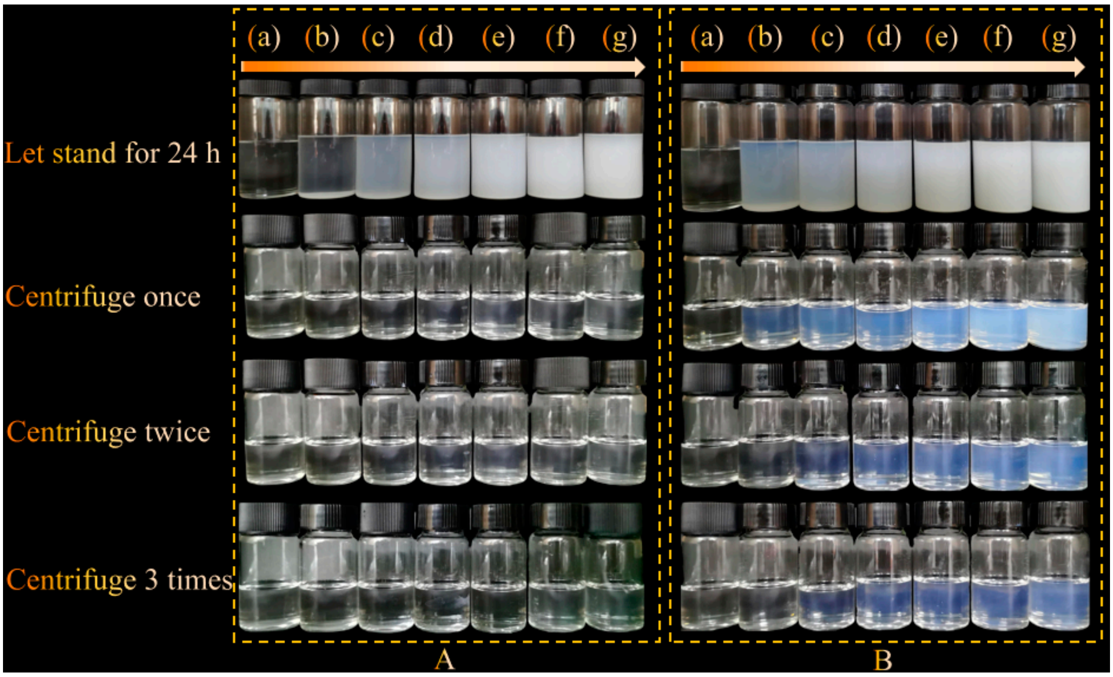

Figure S2 Photographs of DEHC-ZnO (A) and DEHC-PGPR@ZnO (B) after treatment with 24-hour static incubation, one centrifugation, two centrifugations, and three centrifugations, respectively. (a-g) Different ZnO addition concentrations: (a) 0 mg/mL; (b) 0.5 mg/mL; (c) 1.0 mg/mL; (d) 2.5 mg/mL; (e) 5 mg/mL; (f) 10 mg/mL; (g) 15 mg/mL.

After the addition of the PGPR@ZnO ultra-dispersant, as shown in **Figure S2B**, the nanofluid remained stable after standing for 24 h. After high-speed centrifugation, the color was still clearly visible and gradually deepened with increasing ZnO concentration. As shown in **Table S2, Figure S3(b) and (d)**, the residual amounts of ZnO in the fluid almost overlapped after three cycles of centrifugation, indicating that the introduction of PGPR as an ultra-dispersant improved the dispersion stability of ZnO in DEHC. PGPR had been adsorbed onto the surface of ZnO nanoparticles to form an amphiphilic layer, which significantly reduced the water-oil interfacial tension and thus enhanced the dispersibility in

the oil-phase system.[2].

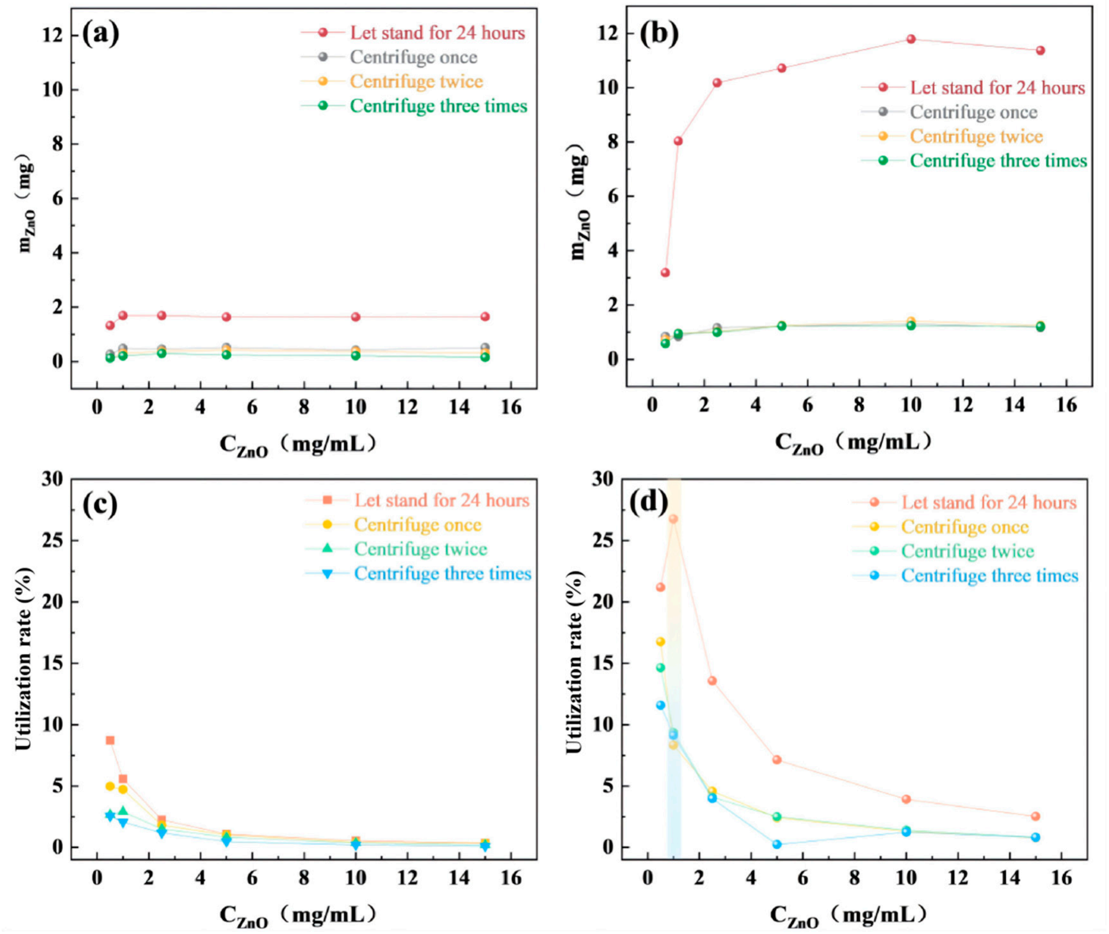

Figure S3 (a-b) Relationship between initial addition concentration and residual amount of ZnO nanoparticles after DEHC-ZnO and DEHC-PGPR@ZnO were left at rest for 24 hours, centrifuged once, centrifuged twice, and centrifuged three times; (c-d) Relationship between the initial addition concentration and utilization rate of ZnO nanoparticles in DEHC-ZnO and DEHC-PGPR@ZnO after 24 hours of standing, one centrifugation, two centrifugations, and three centrifugations.

Table S2. ZnO residual amount and utilization rate at varying addition concentrations and centrifugation cycles

Number of centrifugation	Initial ZnO concentration (mg/mL)	Residual amount of ZnO (mg)	Utilization rate of ZnO (%)	RSD (%)
0	0.5	3.19	21.19%	2.30
	1.0	7.97	26.58%	2.22
	2.5	10.18	13.58%	3.04
	5.0	10.71	7.14%	2.44
	10.0	11.78	3.92%	2.53
	15.0	11.37	2.52%	3.40
1	0.5	0.85	16.77%	2.26
	1.0	0.84	8.34%	3.20
	2.5	1.16	4.58%	2.26
	5.0	1.22	2.43%	3.75
	10.0	1.30	1.30%	2.00
	15.0	1.17	0.78%	2.52
2	0.5	0.73	14.64%	2.52
	1.0	0.96	9.37%	2.65
	2.5	1.02	4.10%	3.27
	5.0	1.25	2.50%	3.75
	10.0	1.40	1.40%	3.00
	15.0	1.25	0.83%	2.78
3	0.5	0.58	11.58%	2.30
	1.0	0.95	9.12%	2.97
	2.5	0.99	3.97%	2.25
	5.0	1.22	0.24%	3.22
	10.0	1.24	1.24%	2.16
	15.0	1.21	0.81%	2.44

References:

1. Suganthi, K. S.; Anusha, N.; Rajan, K. S., Low viscous ZnO–propylene glycol nanofluid: a potential coolant candidate. Journal of Nanoparticle Research *15* (2013), 1986.10.1007/s11051-013-1986-6.
2. Márquez, A. L.; Medrano, A.; Panizzolo, L. A., et al., Effect of calcium salts and surfactant concentration on the stability of water-in-oil (w/o) emulsions prepared with polyglycerol polyricinoleate. Journal of Colloid and Interface Science *341* (2010), 101-108.https://doi.org/10.1016/j.jcis.2009.09.020.
